# Nrf2: not “lost in translation”

**DOI:** 10.18632/aging.101152

**Published:** 2016-12-28

**Authors:** Robert G. Hawley, Irene Riz

**Affiliations:** Department of Anatomy and Regenerative Biology, George Washington University, Washington, DC 20052, USA

**Keywords:** multiple myeloma, carfilzomib, NFE2L2/Nrf2

Multiple myeloma (MM) is a clonal plasma cell malignancy which is frequently diagnosed in patients over 65 years of age. Because of an aging population, the incidence of MM has increased nearly 1 percent annually since 1975 and it is anticipated that the number of cases will nearly double by 2034 [1]. The introduction of novel agents such as the proteasome inhibitor bortezomib (BTZ) has significantly improved overall survival of patients with MM during the past decade. Therapeutic efficacy is related to the exquisite dependence of MM cells on proteasomal degradation of unfolded proteins to maintain proteostasis. However, progression towards BTZ-refractory disease occurs in the majority of MM patients. Unfortunately, once MM patients become refractory to BTZ, their median overall survival has been reported to be less than 1 year [2]. Moreover, in a pivotal phase 2 study with the second-generation proteasome inhibitor carfilzomib (CFZ), less than a 25% response rate was achieved in BTZ-treated patients who had relapsed [3]. These results indicate that the majority of MM cells that became resistant to BTZ were also resistant to CFZ. Clearly, to extend the life expectancy of patients with MM, it is essential to elucidate the underlying mechanisms of acquired proteasome inhibitor resistance.

Toward this goal, we have endeavored to establish clinically relevant CFZ-resistant sublines of patient-derived MM cell lines. We have demonstrated that diverse adaptive mechanisms confer CFZ resistance in these MM cell line models, including increased prosurvival autophagy (as a compensatory mechanism to counter proteasome insufficiency), antioxidant defense and expression of the P-glycoprotein efflux pump [4–6]. In our most recent publication, we described the establishment of a new CFZ-resistant derivative of the LP-1 MM cell line, LP-1/Cfz, in which CFZ resistance involved post-transcriptional activation of nuclear factor-erythroid 2 (NF-E2)-related factor 2 (Nrf2; gene symbol *NFE2L2*) [6]. Under physiological conditions, transcription factor Nrf2 maintains cellular redox homeostasis [7]. Congruent with Nrf2 activation, LP-1/Cfz cells had decreased levels of reactive oxygen species as well as elevated levels of fatty acid oxidation and prosurvival autophagy. Accordingly, genetic and pharmacologic inhibition of Nrf2, disruption of redox homeostasis or inhibition of fatty acid oxidation or autophagy conferred sensitivity to CFZ.

Aberrant Nrf2 pathway activation in LP-1/Cfz cells was associated with elevated levels of sequestosome 1/p62 (SQSTM1/p62). SQSTM1/p62 is a multifunctional scaffold protein which, in addition to activating Nrf2, interacts with various signaling molecules and serves as a ubiquitin-binding cargo receptor connecting the proteasomal and autophagic protein degradation pathways. The higher levels of SQSTM1/p62 were due to increased translation dependent in part on activation of the PERK protein kinase. PERK is normally activated upon accumulation of unfolded proteins in the endoplasmic reticulum which results in the induction of an “unfolded protein response” (UPR). The best characterized function of PERK during UPR induction is to provide a protective advantage to the cell by attenuating global protein translation via inhibitory phosphorylation of eukaryotic translation initiation factor-2α (eIF2α). Phosphorylation of eIF2α also results in the preferential translation of certain mRNAs containing upstream open reading frames, the prototypical example of which is activating transcription factor 4 (ATF4). The data support the view that SQSTM1/p62 translation occurs in part via an ATF4-like mechanism.

PERK also phosphorylates Nrf2 resulting in its activation. Unexpectedly, gene set enrichment analysis (GSEA) indicated that UPR signaling was attenuated in LP-1/Cfz cells. However, a noncanonical mechanism of PERK-Nrf2 activation in the absence of an endoplasmic reticulum stress response has recently been described which results from an epithelial-to-mesenchymal transition (EMT). Along these lines, enrichment of an EMT-like expression signature was indicated by GSEA in LP-1/Cfz cells, and decreased cell surface expression of E-cadherin compared to parental LP-1 cells is consistent with this as a contributory mechanism.

**Figure 1:**
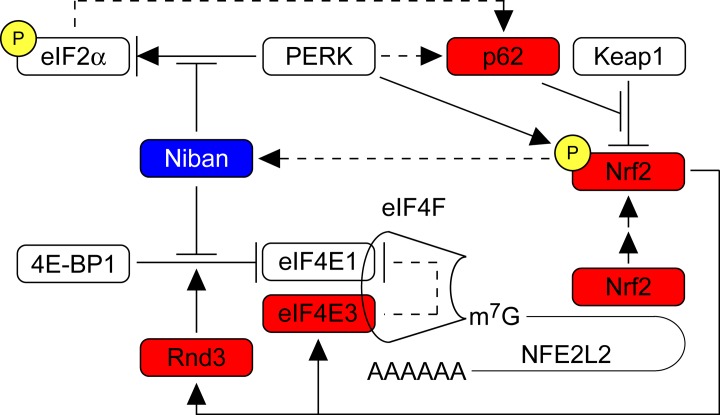
Proposed model of translational reprogramming of the SQSTM1/p62-Nrf2 axis contributing to CFZ resistance in LP-1/Cfz MM cells. Induction of Nrf2 target genes *EIF4E3* (encoding eIF4E3) and *RND3* (encoding Rnd3/RhoE) in LP-1/Cfz cells creates a positive feedback loop promoting eIF4E3-driven Nrf2 translation. *FAM129A* (encoding Niban) is a putative indirect Nrf2 target downregulated in LP-1/Cfz cells. Niban inhibits PERK-mediated phosphorylation of eIF2α. Additionally, Niban (negatively) and Rnd3 (positively) modulate the activity of 4E-BP1, an inhibitor of eIF4E1. NFE2L2 mRNA (encoding Nrf2) is indicated with a 5′-terminal 7-methyl guanosine cap (m^7^G) and a 3′-terminal poly(A) tail (AAAAAA). eIF4F, eukaryotic translation initiation complex containing eIF4E1, eIF4E3 and other components involved in cap-dependent translation initiation; red boxes, increased protein levels; blue box, decreased protein levels; P, phosphorylated form; arrows, positive regulation; bars, negative regulation; dotted lines, undetermined mechanisms. See ref. [6] for details.

Notably, LP-1/Cfz cells exhibited increased translation of Nrf2 which was associated with elevated expression of Nrf2 targets involved in various facets of translational control, in particular, *EIF4E3* encoding a variant component of the eIF4F eukaryotic translation initiation complex. These observations suggested existence of a positive feedback loop. In line with this, we found that siRNA knockdown of EIF4E3 mRNA decreased Nrf2 protein levels. These novel results are summarized schematically in Figure 1.

We demonstrated clinical relevance of our findings by analyzing publicly available gene expression datasets of MM patients with chemoresistant minimal residual disease (MRD) and those whose disease had relapsed. We found that *EIF4E3* expression was increased in the majority of MRD cases in the GEM2010MAS65 clinical trial (GEO accession number GSE70399). Likewise, *EIF4E3* expression was increased in a number of cases of relapsed disease (GEO accession number GSE36824). When GSEA was applied to these cases, significant upregulation of Nrf2 target genes was observed and, as was found for LP-1/Cfz cells, UPR signaling was attenuated.

The data thus suggest that therapies targeting the SQSTM1/p62-Nrf2 pathway may overcome proteasome inhibitor resistance in a subgroup of advanced stage MM patients.
